# A health dialogue intervention reduces cardiovascular risk factor levels: a population based randomised controlled trial in Swedish primary care setting with 1-year follow-up

**DOI:** 10.1186/s12889-017-4670-4

**Published:** 2017-08-22

**Authors:** Mats Hellstrand, Bo Simonsson, Sevek Engström, Kent W. Nilsson, Anu Molarius

**Affiliations:** 1 Competence Centre for Health, Region Västmanland, 721 89 Västerås, Sweden; 2 Department of Public Health and Caring Sciences, Family Medicine and Preventive Medicine Section, Uppsala University, Uppsala, Sweden; 3 Centre for Clinical Research, Region Västmanland /University of Uppsala, Uppsala, Sweden; 4 Karlstad University, Department of Public Health, Karlstad, Sweden

**Keywords:** Health dialogue, Primary care, Life style, Risk factors, Health promotion, Primary prevention

## Abstract

**Background:**

The total number of cardiovascular (CVD) deaths accounted for almost a third of all deaths globally in 2013. Population based randomised controlled trials, managed within primary care, on CVD risk factor interventions are scarce. The aim of the study was to evaluate the effects of a health dialogue intervention in a primary care setting offered to a population at the age of 55 years, focusing on CVD risk factors.

**Methods:**

The study was performed in five primary health care centres in the county of Västmanland, Sweden between April 2011 and December 2012. Men and women were randomly assigned to intervention (*n* = 440) and control groups (*n* = 440). At baseline, both groups filled in a health questionnaire and serum cholesterol, fasting plasma glucose, glycated haemoglobin (HbA1c), weight, height, waist (WC) and hip circumference, waist hip ratio (WHR) and systolic/diastolic blood pressure were measured. Intervention group attended a health dialogue, supported by a visualised health profile, with a possibility for further activities. Participation rates at baseline were 53% and 52% respectively. A 1-year follow-up was carried out.

**Results:**

The intervention group (*n* = 165) showed reductions compared to the control group (*n* = 177) concerning body mass index (BMI) (0.3 kg/m^2^, *p* = .031), WC (2.1 cm, *p* ≤ .001) and WHR (.002, *p* ≤ .001) at the 1-year follow-up. No differences between the intervention and control groups were found in other variables. Intervention group, compared to baseline, had reduced weight, BMI, WC, WHR, HbA1c, and diet, while the men in the control group had reduced their alcohol consumption.

**Conclusions:**

A health dialogue intervention at the age of 55 years, conducted in ordinary primary care, showed a moderate effect on CVD risk factor levels, in terms of BMI, WC and WHR.

**Trial registration number:**

BioMed Central, ISRCTN22586871, date assigned; 10/12/2015

## Background

The total number of cardiovascular deaths accounted for almost a third of all deaths globally in 2013 [1]. In high and middle income countries cardiovascular disease (CVD) is the most common cause of mortality although its occurrence has decreased since 1990s [2]. CVD is a major public health problem in Sweden [3] and it’s incidence in Västmanland county is similar to the rest of the country [4].

In order to explore the risk factors predisposing the development of CVD, the Framingham study was initiated in 1948 and is still continuing [5]. Since then several CVD-focused population based intervention programs have been developed in the Nordic countries [6–11] and elsewhere [12]. In Sweden, the Sollentuna primary care prevention program [13] reported a reduction of acute myocardial infarction incidence among women and the Västerbotten CVD Intervention Program [14] reported a lower all-cause mortality. On the other hand, the Inter99 randomised controlled trial, carried out in Copenhagen, Denmark, [15] found no significant population effects for ischaemic heart disease, stroke or mortality at 10-year follow-up, but sustained effects on physical activity, diet, smoking and alcohol consumption for the intervention group [16, 17].

Moreover, significantly reduced cardiovascular risk scores, body mass index (BMI) and serum cholesterol levels (S cholesterol) in the intervention groups compared to the control group were found at 5-year follow-up in the randomised controlled study in Ebeltoft, Denmark, but only for the health screening intervention and not for the health dialogue intervention [18, 19]. In Skaraborg, Sweden, significant effects were found on dietary habits, BMI, waist circumference (WC), S cholesterol, systolic/diastolic blood pressure (SBP/DBP), and metabolic risk profile when comparing four intervention communities with reference communities [20]. The intervention consisted of an individual health dialogue combined with a global health and risk assessment tool, the Habo Health Curve [7, 8, 20]. A 1-year follow-up after a life style intervention in primary care in Hisingen, Gothenburg, showed reduced risk scores [21]. Positive short term effects on life style factors at 1-year follow-up have also been published from Canada [22]. While smoking prevalences are decreasing in most Western countries [23], obesity is increasing [24], indicating a need for improvements concerning diet and physical activity in these populations. Population based randomised controlled trials, managed within primary care, and with specific focus on the effects of a health dialogue have to our knowledge not been conducted.

The primary aim of this study was to explore the effects of a health dialogue intervention on CVD risk factors, comparing an intervention group with a control group. A secondary aim was to compare an intervention subgroup at elevated risk with a control group at elevated risk, in order to explore the effects of the health dialogue intervention for those in need for change.

## Methods

### Study design

The study was a population based randomised controlled trial in a Swedish primary care setting, aimed at studying the effects of a health dialogue intervention on CVD risk factor levels. Ethical approval for the study was received from Uppsala Ethical Review Board in December 2010 (Dnr 2010/427).

The study was performed in five primary health care centres in the county of Västmanland between April 2011 and December 2012. Three of them are located in Västerås and the other two in small towns. The five centres cover a population with a socioeconomic variation similar to the general population of Västmanland county and Sweden. Agreements were signed with each centre stipulating activities, personnel and economic resources for their participation. Activities consisted of counseling and administration time for nurses, as well as laboratory and medical resources. Medical treatment was initiated when needed due to test results and considered as ordinary care. One centre had three nurses involved, three centres had two nurses and the smallest centre had one nurse. The nurses were trained for 2 days in the method. Within the primary care centres there were no local coordinator or other project supporting resources.

The computerised randomisation was made by a research assistant with no other involvement in the present study, at the Centre of Clinical Research, Västerås Hospital. Swedish primary care centres deliver health care to those inhabitants who are listed at that centre. All inhabitants in Sweden are listed at a primary care centre. 880 persons born in 1956–1957 and listed at the five primary care centres were invited to participate in the study, with no exclusion for chronic disease, medication or other medical conditions and simple randomisation procedure was conducted within each centre population before the invitation. This procedure was carried out from the listing register. Each individual received a written information about the aim of the study and specified activities and an invitation to participate. If he/she decided to participate, a written informed consent was obtained.

### Participants and intervention

**Fig. 1:**
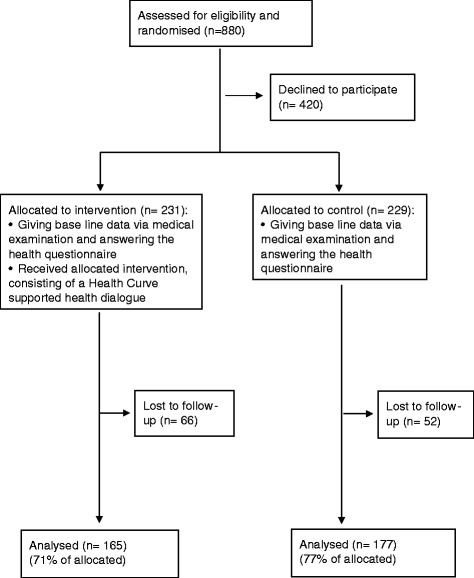
Flow chart

The study population was assigned to intervention (*n* = 440) and control (*n* = 440) groups (Fig. 1). 231 (53%) of those randomised to the intervention group participated at the baseline, compared to 229 (52%) in the control group. At baseline, the intervention and control groups filled in the Habo Health Curve questionnaire [7, 8, 20] and had their blood tests taken. The intervention group attended a health dialogue supported by a visualised health profile, the Habo Health Curve [7, 8, 20]. The Habo Health Curve covers the following risk and health factors for CVD: tobacco, alcohol, diet, physical activity, psychosocial strain, mental stress, BMI, waist hip ratio (WHR), S cholesterol, SBP/DBP, chronic disease, heredity for diabetes, diabetes and cardiovascular disease. Scientific background, method for measurement of each risk factor and transferring them into the Habo Health Curve is described elsewhere [8].

The health dialogue in the intervention group consisted of motivational interviewing [25–27] and structured counseling for 60–75 min. The participant and the primary care nurse monitored the participant’s health profile based on the results of the tests and the questionnaire. Risk factors and their interrelations were discussed. The dialogue also included a discussion about how to best decrease the risk. The graphical health curve was seen as an educational tool during this dialogue. When considered appropriate by the participant, the nurse arranged additional support, following recommendations in a special manual for the Habo Health Curve method. 15% of the intervention group (*n* = 25) wanted and received such additional motivational support for life style changes, without medical treatment and for another 9% (*n* = 14) this was combined with a consultation about medical treatment. So altogether 24% (*n* = 39) received additional support for life style changes.

During the study, information about each participant group allocation was available for the health care providers, i.e. the study was not blinded. Baseline visits for medical tests and health dialogue were carried out from April to December 2011, proportionally per month.

When the control group participants were found having diverging medical test results, the primary care centre was informed and initiated usual care routines, otherwise the control group participants received a letter informing that the test results were normal. For intervention group participants, both diverging and not diverging test results were presented and discussed in the health dialogue.

A 1-year follow-up was conducted for intervention (*n* = 165) and control groups (*n* = 177) using the same methodology as at baseline (Fig. 1). In order to minimise possible bias from seasonal variation the 1-year follow-up was carried out 11–13 months after the baseline. 29% of the intervention group (*n* = 52) and 23% of the control group (*n* = 52) were lost to follow-up, due to declining the invitation or not responding.

Sample size calculation defined that a sample size of 440 individuals, with margin for dropout, per intervention and control groups was necessary to detect a difference in change in BMI of 0.35 kg/m^2^, (SD 1.5) with a two-sided 5% significance level and a power of 80%.

### Measurements

The following biological variables were measured: weight, height, WC, WHR, SBP/DBP,

S cholesterol, glycated hemoglobin (HbA1c) and fasting plasma glucose (Fp glucose). Self reported variables included physical activity, alcohol consumption last week, diet and smoking habits, defined as in the Habo Health Curve questionnaire [7, 8, 20]. Physical activity was measured through the 4-level Saltin-Grimby Physical Activity Level Scale [28]. The dietary questionnaire was based on 20 questions and has proved to be useful for estimating semi quantitatively the intake of dietary fat and the proportion of fibre in the diet [29]. Risk points were based on the points estimated from fat and fibre intake.

Blood pressure was measured manually in the right arm in sitting position after 5 minutes rest. Weight was measured with light clothing and without shoes, while height was measured without shoes on a fixed wall measure, to the nearest centimetre. BMI (kg/m^2^) was calculated as weight/ (height x height). WC was measured in the standing position at the level midway between the lower rib margin and the iliac crest. Hip circumference (HC) was measured as centimetre at the widest point between hip and buttock. WHR was calculated as WC divided by HC (cm/cm).

Blood tests were taken venous on fasting participants at the primary care centres. The central laboratory at Västerås Hospital, authorised according to ISO 17025, carried out the analyses in the study. Device for lipid analyses was AU680 from Beckman Coulter and the analyses, from both baseline and 1 year follow-up, were carried out in September 2013 after storage of tests in −70 degrees Celsius. Glucose analyses were carried out using FC Mixture-tubes (EDTA-Na2 + Citrat + NaFlourid) and glycated hemoglobin was analysed using Tosoh Automated Glucohemoglobin Analysator HLC-723G7. All analyses were destroyed at the end of the study.

Extreme values were excluded from the analysis due to report error. Two values concerning WC were excluded, one with a reduction of 30 cm (weight difference; − 2.0 kg) and one with 35 cm (weight difference; −2.1 kg). Two values concerning alcohol consumption were excluded as extreme, one with an increase of 226 cl 40% liquor equivalents, and one with 194 cl.

### Statistical analyses

Statistical analyses were performed using SPSS, version 20.0. Continuous variables were analysed with independent samples T-test between groups at baseline and at 1 year follow-up. Paired samples T-test was used within groups between baseline and 1 year follow-up. Categorical values at baseline were analysed with Pearson Chi Square test. McNemar test was used to assess change in categorical variables within groups. Analyses were conducted at 1-year follow-up for each variable.

Group characteristics at baseline in intervention and control groups were analysed. Between groups differences in CVD risk factor changes at 1-year follow-up were calculated to answer the main aim of the study. In addition, differences in risk factor changes were studied within the intervention and control groups.

Analyses of risk factor changes were conducted also for subgroups with elevated baseline levels, in order to explore effects among those with need for improvements. Definition of elevated risk factor levels, was BMI ≥27 kg/m^2^ for men and BMI ≥29 kg/m^2^ for women, WHR ≥0.95 for men and ≥0.83 for women, S cholesterol ≥5.0 mmol/l, SBP ≥ 140 mmHg, alcohol ≥25 cl 40% liquor equivalents/week for men and ≥17 cl for women, inactive or light physical activity following a four degree scale, diet points ≥ 6 following an eleven degree scale. All these cut offs were defined as in the Habo Health Curve [7, 8, 20] except for physical activity which was defined through the 4-level Saltin-Grimby Physical Activity Level Scale [28].

In order to assess possible bias in the results due to loss of follow-up, analyses of baseline variables were carried out comparing those completing the study and those participating at baseline but lost to follow-up.

Effect sizes of statistically significant between group differences were measured [30]. For this purpose Cohen’s *d* values [31] were calculated.

## Results

### Baseline values

Baseline values for participants who completed the study are described in Table 1. Intervention and control groups had no statistically significant differences at baseline, except for a difference between women in intervention and control groups concerning DBP (*p* = .02). More women than men participated in the intervention group, whereas there was no difference in the control group.

**Table 1:** Base line values on CVD risk factors (2011)

	Intervention						Control						Difference
	Men		Women		Total		Men		Women		Total		Intervention-Control
Continuous variables	Mean (SD)	n	Mean (SD)	n	Mean (SD)	n	Mean (SD)	n	Mean (SD)	n	Mean (SD)	n	*p-*value
Weight (kg)	91.3 (14.9)	67	72.4 (13.2)	89	80.5 (16.8)	156	90.4 (12.4)	72	72.9 (12.2)	75	81.4 (15.1)	147	.599
BMI (kg/m2)	27.98 (3.6)	67	26.71 (4.9)	89	27.26 (4.4)	156	27.83 (3.2)	72	26.47 (4.1)	75	27.14 (3.8)	147	.795
WC (cm)	103.4 (11.5)	68	92.6 (12.3)	90	97.3 (13.1)	158	101.1 (8.8)	72	91.4 (10.3)	75	96.2 (10.7)	147	.422
HC (cm)	106.9 (6.6)	68	105.6 (9.2)	90	106.1 (8.2)	158	106.6 (6.3)	72	106.0 (8.3)	76	106.3 (7.4)	148	.872
WHR	0.97 (0.07)	68	0.88 (0.07)	90	0.91 (0.08)	158	0.95 (0.05)	71	0.86 (0.06)	75	0.90 (0.07)	146	.198
SBP (mm Hg)	136.8 (13.6)	68	127.8 (15.3)	90	131.6 (15.2)	158	133.1 (15.7)	73	132.1 (17.5)	76	132.6 (16.6)	149	.605
DBP (mm Hg)	86.7 (9.0)	68	79.0 (8.7)	90	82.3 (9.6)	158	85.0 (10.2)	73	82.2 (8.9)	76	83.5 (9.6)	149	.258
Cholesterol (mmol/L)	5.6 (1.0)	41	6.3 (1.2)	48	6.0 (1.2)	89	5.8 (1.1)	57	6.5 (1.1)	55	6.1 (1.2)	112	.401
HbA1c (mmol/mol)	38.2 (7.7)	70	36.7 (4.1)	95	37.4 (5.9)	165	37.9 (10.3)	85	37.0 (3.4)	88	37.4 (7.6)	173	.893
Fp glucose (mmol/L)	6.3 (1.4)	68	5.6 (0.6)	88	5.9 (1.1)	156	6.3 (1.4)	79	5.7 (0.67)	83	6.0 (0.9)	162	.347
Alcohol consumption^a^	38.3 (31.3)	61	20.3 (19.7)	57	29.6 (27.8)	118	42.8 (35.2)	79	21.0 (18.9)	68	32.7 (30.7)	147	.398
Categorical variables	%		%		%		%		%		%		
Low physical activity^b^	83.3	66	73.9	88	77.9	154	75.0	84	74.7	87	74.9	171	.516
Unfavourable diet^c^	69.2	52	43.1	65	54.7	117	74.6	67	28.2	71	50.7	138	.526
Daily smokers	7.4	68	12.6	87	10.3	155	10.8	83	11.8	85	11.3	168	.776

^a^Cl as 40% liquor equivalents last week

^b^No intense physical activity last week

^c^Diet points > 5 of 11, summarising the level of fibre intake and saturated fat intake last week

Variation in number of subjects per variable is due to internal data loss.

At baseline 33% (*n* = 54) of the intervention group reported high blood pressure diagnosed by a doctor, compared to 28% (*n* = 49) of the control group. 24% (*n* = 39) in the intervention group had medication for high blood pressure, compared to 19% (*n* = 34) in the control group. In the intervention group 5% (*n* = 9) reported diabetes diagnosed by a doctor and 3% (*n* = 6) in the control group. Participants with diverging test results in intervention (*n* = 21) and control groups (*n* = 17) were referred to medical care.

### Between group differences

Between group differences in CVD-related factors at 1-year follow-up (2012) compared to baseline are described in Table 2.

**Table 2:** Changes in CVD-related factors at one year follow-up (2012) compared to baseline^a^

	Intervention						Control						Between groups		Effect size
	Men		Women		Total		Men		Women		Total				
Continuous variables	Mean (SD)	*p*-value	Mean (SD)	*p*-value	Mean (SD)	*p*-value	Mean (SD)	*p*-value	Mean (SD)	*p*-value	Mean (SD)	*p*-value	Mean (SEdiff)	*p*-value	
Weight (kg)	−0.6 (3.7)	.181	−0.9 (3.3)	.009	−0.8 (3.4)	.005	−0.1 (3.0)	.773	−0.21 (3.2)	.571	−0.2 (3.1)	.538	0.6 (0.38)	.093	
BMI (kg/m^2^)	−0.22 (1.1)	.132	−0.44 (1.3)	.002	−0.35 (1.2)	.001	−0.08 (1.1)	.548	−0.02 (1.2)	.897	−0.05 (1.2)	.622	0.31 (0.14)	.031	.25
WC (cm)	−2.3 (5.6)	.001	−1.0 (4.7)	.057	−1.5 (5.1)	≤ .001	−0.2 (4.0)	.720	1.4 (5.1)	.019	0.6 (4.7)	.105	2.1 (0.56)	≤ .001	.44
WHR	−0.02 (0.1)	.009	0.00 (0.1)	.380	−0.01 (0.1)	.013	0.00 (0.0)	.665	0.01 (0.1)	.018	0.01 (0.0)	.034	0.02 (0.01)	≤ .001	.38
SBP (mm Hg)	−2.6 (13.7)	.121	−0.7 (13.3)	.636	−1.5 (13.5)	.163	−1.0 (13.9)	.541	−0.95 (14.8)	.578	−1.0 (14.3)	.408	1.00 (1.59)	.740	
DBP (mm Hg)	−2.1 (9.6)	.073	1.2 (8.8)	.215	−0.3 (9.3)	.731	−0.5 (10.4)	.669	0.47 (7.3)	.572	0.0 (8.9)	.985	0.3 (1.04)	.817	
S-cholesterol (mmol/L)	−0.2 (0.8)	.095	−0.2 (1.2)	.341	−0.2 (1.1)	.085	−0.1 (0.9)	.412	−0.1 (1.0)	.322	−0.1 (0.9)	.196	0.1 (0.14)	.574	
HbA1c (mmol/mol)	−0.3 (2.9)	.430	−0.6 (2.2)	.006	−0.5 (2.5)	.013	−0.5 (7.1)	.506	0.1 (1.9)	.528	−0.3 (5.2)	.419	−0.17 (0.44)	.698	
Fp glucose (mmol/L)	0.1 (0.8)	.610	0.0 (0.4)	.980	0.0 (0.6)	.668	−0.2 (0.8)	.071	0.0 (0.5)	.569	−0.1 (0.7)	.065	−0.1 (0.07)	.102	
Alcohol consumption	−0.5 (22.9)	.857	−1.8 (12.0)	.365	−1.0 (18.4)	.566	−5.3 (19.9)	.021	−2.6 (13.3)	.117	−4.0 (17.2)	.005	−3.0 (2.19)	.167	
Categorical variables	%	*p*-value	%	*p*-value	*%*	*p*-value	%	*p*-value	%	*p*-value	%	*p*-value			
Unfavourable diet	0.0	1.000	−20.0	.004	−11.1	.031	−6.0	.424	1.8	.815	−0.6	.860			
Low physical activity	−7.5	.180	−5.7	.267	−6.5	.052	−3.5	.549	1.2	1.000	−1.2	.855			
Daily smokers	1.5	1.000	2.3	.625	−0.6	1.000	0.0	1.000	0.0	1.000	0.0	1.000			

^a^n-values are the same as in Table 1

The intervention group showed statistically significant improvements compared to the control group for BMI (0.31 kg/m^2^, Cohen’s *d* = .25), WC (2.1 cm, Cohen’s *d* = .44) and WHR (0.02, Cohen’s *d* = .38), (Table 2).

Men in the intervention group had statistically significant improvements compared to men in the control group, concerning WC and WHR. Among the women, there were statistically significant group differences in favour of the intervention group for BMI, WC and WHR.

### Within group differences

Intervention group had significantly reduced weight, BMI, WC, WHR, and HbA1c and more favourable diet. The increase in physical activity was of borderline significance. The control group had increased WHR among women, and reduced alcohol consumption among men at 1-year follow-up (Table 2).

### Analysis in the subgroup with elevated risk at baseline

Analysis of the subgroup with elevated risk was performed for within and between group differences, as shown in Table 3.

**Table 3:** Changes in CVD-related factors at one year follow-up (2012) among participants with elevated risk at baseline

	Intervention			Control			Between group differences		Cohen’s d
Continuous variables	Mean (SD)	*p*-value	n=	Mean (SD)	*p*-value	n=	Mean (95% CI)	*p*-value	
Weight (kg)	- 1.6 (4.1)	.004	59	- 0.1 (3.8)	.817	62	1.49 (0.7, 2.9)	.039	.38
BMI (kg/m^2^)	- .62 (1.44)	.002	59	- 0.10 (1.5)	.585	62	.52 (0.0, 1.0)	.052	
WC (cm)	- 2.82 (5.5)	≤ .001	99	- 0.1 (4.7)	.892	89	2.75 (1.3, 4.2)	≤ .001	.54
WHR	- .025 (0.1)	≤ .001	99	- 0.00 (0.0)	.938	89	.03 (0.0, 0.0)	≤ .001	.52
S-cholesterol	- .29 (1.0)	.015	72	- 0.16 (1.0)	.109	95	.13 (−0.2, 0.4)	.398	
SBP	7.04 (14.1)	≤ .001	52	−7.19 (17.6)	.005	52	.15 (−6.4, 6.0)	.961	
Alcohol consumption	- 4.7 (22.1)	.090	67	- 7.7 (19.9)	≤ .001	89	3.05 (−9.7, 3.6)	.367	
Categorical variables	%			%					
Unfavourable diet	−34.4	≤ .001	64	−24.3	≤ .001	70			
Low physical activity	−13.3	≤ .001	120	−12.5	≤ .001	128			

Analysis in the subgroups with elevated risk at baseline showed statistically significant effects in weight, WC and WHR between intervention and control groups. Furthermore, the intervention high risk group showed decreased proportions with unfavourable diet and low physical activity. The control group with unfavourable risk factor levels had no improvements at 1-year follow-up on weight, BMI, WC and WHR. Both intervention and control groups with elevated risk showed decreased SBP.

### Lost to follow-up

Analyses of baseline variables were carried out, with comparison between those completing the study and those participating at baseline but lost to follow-up. The group lost to follow-up had a significantly higher alcohol consumption compared to the group completing the study. No other differences between the groups were found (data not shown).

## Discussion

### Main results

The health dialogue intervention reduced some of the CVD risk factors. The decrease in the intervention group compared to the control group was statistically significant at 1-year follow-up for BMI, WC and WHR. In addition, weight, HbA1c and unfavourable diet were significantly reduced in the intervention group.

Similar effects have previously been found at 5-year follow up in the Ebeltoft study [18, 19] and in the Lingfors et al. [20] study, and also at 1-year follow-up in the Blomstrand et al. [21] and the Cox et al. [22] studies. The Blomstrand et al. study, recruiting patients visiting primary care centres, included components such as answering a health questionnaire, undergoing medical tests, participating in a health dialogue and being referred to medical care when needed. Our study, being population based, showed stronger effects than Blomstrand et al. on weight, BMI and waist. However, also the Blomstrand study reported significant effects, due to a larger sample size (*n* = 2120). The Cox et al. study included patients with high or medium risk and had large effects on CVD risk factors. The present study results suggest that there are beneficial effects for the total population at the age of 55 as for the unselected patient groups in the Blomstrand et al. study. Our study did not show effects in the cardiovascular risk score as was found in the Ebeltoft study, since there were no statistically significant effects on SBP/DBP, S cholesterol or smoking prevalence. However, our study found a statistically significant effect on BMI.

In our study, the difference in the decrease between intervention and control groups was 0.3 kg/m^2^ BMI and 2.1 cm for WC which can be seen as moderate. The effect sizes, measured with Cohen’s *d,* ranged from 0.25 to 0.44 and can be interpreted as being from small to medium [30]. Such a classification is though somewhat arbitrary and depends on the context [32]. The preventive paradox [33] denotes that interventions targeting the general population, aiming at shifting the risk curve to the left, are more effective for public health than interventions targeting high risk groups. Even clinically small effects can therefore be of interest from public health perspective [34]. In our study, the analysis of the subgroup with elevated risk at baseline showed that the health dialogue intervention was effective also among those who most needed improvements, even though the number of subjects in our study was limited. The intervention can thus be seen as a combination of a population and a high risk strategy. The method contains a low dose of intervention, applicable in ordinary primary care. Such interventions are needed to tackle the increasing obesity rates in most Western populations [24].

The Inter99 trial [15] found no significant population effect at 10-year follow-up on ischaemic heart disease, stroke, combined events or mortality, but sustained effects on risk factors [16, 17]. The Sollentuna and Västerbotten primary care integrated CVD intervention studies showed, however, significant population effects during 15–20 year follow-up on acute myocardial infarction and all-cause mortality respectively [13, 14]. The present study was primary care integrated and applied a visualised supported health dialogue similar to the Västerbotten study. This increases the comparability of our study results, highlighting the specific life style effects of a health dialogue as one central component of the intervention. This is especially interesting since it has been shown that life style and biological markers can predict morbidity and mortality up to 26 years of follow-up [35].

### Strengths and limitations

A major strength of our study is that it was a randomised controlled trial. Another strength is the setting within primary care, which supports the applicability of the study results. The effects of the health dialogue intervention are supported by analysis of internal data loss between baseline and 1-year follow-up, showing similar proportionate data loss in intervention and control groups, except for alcohol consumption. In addition, the study results gather around a coherent set of risk factor values. The risk for random significances due to multiple tests can therefore be regarded as small [36, 37].

The present study had limited resources and a lower participation rate than planned, which generated restricted power to detect effects of the intervention. In spite of this, statistically significant effects on BMI, WC and WHR were found. Participants in both the intervention and control groups underwent standard medical treatment if they had diverging laboratory results. Therefore, there was limited room to detect an effect in laboratory results. The secular trend in mid-Sweden showed increasing prevalence of obesity in the population of 25–74 years from 12% in year 2000 to 17% in year 2012, for both men and women [38]. Thus, it is unlikely that our results could be explained by a secular trend.

Test results of blood samples analysed after storage at −70 degrees Celsius may diverge from tests analysed directly after collection [39]. As all blood tests in our study were analysed after being stored in −70 degrees Celsius, comparisons within and between groups can be assumed to be valid.

The follow-up was restricted to 1 year, why it is not possible to evaluate long term effects of the intervention. The overall baseline participation rate in the study was lower than expected, 52%. However, similar participation rates were obtained both in the intervention and the control groups. Participation rates of 65–70% have been reported from another population based study in primary care setting [40], while other studies in Sweden have been patient based. It is therefore important to predict probable participation rates when planning similar interventions and take into consideration what is feasible in primary care settings.

## Conclusion

A health dialogue intervention at the age of 55, conducted in ordinary primary care setting, showed a moderate effect on CVD risk factor levels, in terms of BMI, WC and WHR.

## Abbreviations

BMI: Body mass index
CVD: Cardiovascular disease
DBP: Diastolic blood pressure
HbA1c: Glycated haemoglobin
HC: Hip circumference
S cholesterol: Serum cholesterol
SBP: Systolic blood pressure
WC: Waist circumference
WHR: Waist hip ratio
